# Thymidylate synthase expression and p21^WAF1^/p53 phenotype of colon cancers identify patients who may benefit from 5-fluorouracil based therapy

**DOI:** 10.1007/s13402-013-0159-z

**Published:** 2013-11-26

**Authors:** Violetta Sulzyc-Bielicka, Pawel Domagala, Dariusz Bielicki, Krzysztof Safranow, Wenancjusz Domagala

**Affiliations:** 1 Department of Clinical Oncology, Pomeranian Medical University, Szczecin, Poland; 2 Department of Pathology, Pomeranian Medical University, Unii Lubelskiej 1, 71-252 Szczecin, Poland; 3 Department of Gastroenterology, Pomeranian Medical University, Szczecin, Poland; 4 Department of Biochemistry and Medical Chemistry, Pomeranian Medical University, Szczecin, Poland

**Keywords:** Colorectal cancer, p21^WAF1^, p53, Thymidylate synthase, 5FU-based therapy

## Abstract

**Background:**

Studies on the expression of thymidylate synthase (TS) in colorectal cancers (CRCs) have failed to provide unequivocal prognostic or predictive information. Here, we assessed the prognostic significance of TS expression in Astler-Coller stage B2 and C CRCs defined by a p21^WAF1^/p53 immunophenotype in patients subjected to 5-fluorouracil (5FU)-based adjuvant therapy.

**Methods:**

A cohort of 189 CRCs was asssessed for TS, p21^WAF1^ and p53 expression on tissue microarrays using immunohistochemistry, and associations with disease-free survival (DFS) and overall survival (OS) of the patients were assessed using univariate and multivariate analyses.

**Results:**

TS expression led to the stratification of patients with colon cancer, but not rectal cancer, with immunophenotypes other than p21^WAF1^+/p53- (referred to as P&P) into subgroups characterized by a worse (P&P TS+) and a better (P&P TS-) DFS and OS, in univariate (*P* = 0.006 and *P* = 0.005, respectively) and multivariate (*P* = 0.0004 and *P* = 0.002, respectively) analyses. The p21^WAF1^+/p53- immunophenotype was associated with a favorable prognosis, irrespective of TS expression.

**Conclusions:**

The strong association observed between the P&P TS+ immunophenotype and a worse DFS and OS suggests a predictive significance of TS expression for 5FU-based adjuvant therapy in patients with colon cancers exhibiting the P&P immunophenotype. In addition, our findings suggest that the appropriate target for assessment of TS expression as a prognostic/predictive marker is a subgroup of colon cancers with an immunophenotype other than p21^WAF1^+/p53-, and that only in this subgroup high TS expression is associated with an unfavorable DFS and OS. Therefore, we suggest that assessing TS expression in conjunction with p21^WAF1^/p53 immunophenotyping of colon cancers may improve the selection of patients suitable for 5FU-based adjuvant chemotherapy.

## Introduction

Significant progress in the systemic treatment of colorectal cancer (CRC), attributed to the introduction of new drugs, has not affected the efficacy of fluoropyrimidine analogs in chemotherapy regimens where they are used as vehicles to improve therapeutic responses. However, controversies remain concerning indications for adjuvant therapy in stage II CRC pateints. It has been argued that only one out of 25 patients with stage II CRC may be cured by 5-flurouracil (5FU)-based adjuvant chemotherapy [1]. Thus, there is a need to identify predictive markers for 5FU-based chemotherapy responses. The effect of 5FU-based therapy depends on the inhibition of thymidylate synthase (TS), a key enzyme in nucleotide synthesis, and a subsequent p53-dependent induction of apoptosis through cell cycle regulatory proteins [2–4]. This notion, corroborated by in vitro research using cell lines, has led to several clinical trials with the objective to determine the role of TS in 5FU-based adjuvant chemotherapy. The results obtained have, however, been discrepant and disappointing, and have failed to provide unequivocal prognostic or predictive information. Therefore, studies on the expression of TS in CRC have been labeled as a “never-ending story” [5]. One reason for this in limbo situation may be that, with the exception of p53 [6], the expression of important cell cycle regulatory proteins has not been considered in studies aimed at identifying sugroups of patients in which TS could play a role.

Here, we assessed the prognostic significance of TS expression in patients with Astler-Coller stage B2 and C CRCs defined by the p21^WAF1^/p53 immunophenotype and subjected to 5FU-based adjuvant chemotherapy. Our findings suggest that the appropriate target for assessment of TS expression as prognostic/predictive marker is a subgroup of colon cancers with an immunophenotype other than p21^WAF1^+/p53-, and that only in this subgroup high TS expression is associated with an unfavorable DFS and OS.

## Materials and methods

### Patients

The study group consisted of 189 nonselected, consecutive patients who met the following criteria: (1) patients had undergone potentially curative colorectal resection for sporadic CRC (defined as absence of relevant family history at the time of admission to the hospital), (2) distant metastases were excluded upon preoperative liver ultrasonography, chest x-ray, and during intraoperative exploration, (3) patients had no chemotherapy prior to the operation, (4) patients had a histopathologic diagnosis of invasive adenocarcinoma Astler-Coller B2 or C, without involvement of resection margins, and (5) patients received identical adjuvant 5FU-based therapy.

All available histological slides for all patients were re-examined. In Table 1 the clinico-pathological details of the 189 tumors and patients are listed. The mean age of the patients was 59.1 (range 33–81, median 60) years. Tumors were resected at the Departments of Surgery of the Pomeranian Medical University teaching hospitals in Szczecin and the Regional Oncological Center in Szczecin, Poland. The operations consisted of either a resection with lymphadenectomy or a total mesorectal excision for rectal carcinomas. All patients (76 stage B2, and 113 stage C) were treated with the same adjuvant chemotherapy regimen (six 5-day courses of bolus infusions of 5FU [425 mg/m^2^] every four weeks combined with leucovorin 20 mg/m^2^).

**Table 1:** Clinicopathological characteristics of the study group (*n* = 189)

Parameter	n (%)
Age (years)	
≤60	100 (53)
>60	89 (47)
Sex	
Females	87 (46)
Males	102 (54)
Grade	
1 + 2	100 (53)
3^1^	89 (47)
Astler-Coller stage	
B2	76 (40)
C	113 (60)
Site	
Rectum	93 (49)
Colon	96 (51)
Radiotherapy (rectal tumors) (*n* = 74)	
Preoperative	32 (43)
Postoperative	40 (54)
Unknown	2 (3)

^1^Including mucinous carcinoma

Forty out of 93 patients with rectal cancer received postoperative radiotherapy (50.4 Gy) and 32 out of these patients received preoperative radiotherapy (5 × 5 Gy). Of the 76 Astler-Coller stage B2 tumors 36 were rectal tumors and, of these, 15 (42 %) received preoperative and 10 (28 %) postoperative radiotherapy. Since there were no statistically significant differences in TS, p53, and p21^WAF1^ expression between rectal cancers of patients who did or did not undergo preoperative radiotherapy, the former were included in the study [TS (53.1 % vs. 62.7 %, respectively, *P* = 0.38), p53 (65,6 % vs. 69.5 %, respectively, *P* = 0.81), and p21^WAF1^ (53.1 % vs. 66.1 %, respectively, *P* = 0.26)].

The time from surgery until the time of death due to cancer or to last known follow-up was regarded as OS, and the time until the first appearance of a metastasis or a local recurrence was regarded as DFS. The median follow-up was 51 (mean, 51.1 ± 23.8; range, 7–120) months. During the follow-up, 43 of the 189 (22.8 %) patients died of their disease and 115 (60.8 %) were alive without disease symptoms. Recurrences were found in 74 patients. Four patients died of non-cancer related causes and were treated as censored observations.

### Tissue microarray construction

Tumor tissue was fixed in buffered 10 % formalin and embedded in paraffin. Sections (4 μm thick) were stained with hematoxylin and eosin for histopathological diagnosis. Tissue microarrays were constructed as previously described [7]. In short, one 0.6 mm core was taken from a carefully identified, histologically relatively homogenous, representative area with the highest mitotic activity at the outer invasive zone of each CRC.

### Immunohistochemistry

Tissue microarray slides were deparaffinized, rehydrated, and endogenous peroxidase activity was blocked. Slides were immersed in pH 9.0 buffer and heat-induced antigen retrieval was performed in a pressure cooker (Pascal, DakoCytomation). The slides were incubated for 30 min with the following monoclonal antibodies: anti-p21^WAF1^ antibody (dilution 1:25), anti-TS106 antibody (dilution 1:50; Chemicon, Temecula, USA), and anti-p53 antibody (dilution 1:50). The slides were immunostained using a Dako EnVision kit according to the manufacturer’s instructions (EnVision™ + Peroxidase Anti-mouse Polymer labeled with horseradish peroxidase, Dako Co., Carpinteria, CA). We used the sensitive EnVision™ + visualization system, since the detection system used is regarded as a critically important variable in immunohistochemical analysis, and since detection methods using signal amplification with HRP-labeled polymers (such as EnVision™) have been shown to be more sensitive than methods without such a layer of amplification [7]. The reaction was developed with diaminobenzidine substrate-chromogen solution, and the slides were counterstained with hematoxylin. Appropriate positive and negative controls were included. The immunohistochemical procedures for all tissue microarrays encompassing all 189 tumors were performed at the same time under identical conditions. Only four slides containing tissue cores from all 189 tumors were processed.

Immunohistochemistry for assessing the presence or absence of DNA mismatch repair (MMR) proteins was performed on 185 CRCs (4 tumors could not be assessed due to insufficient amount of tissue). Immunohistochemistry with antibodies directed against MMR proteins has been regarded as an equivalent for microsatellite instability (MSI) testing [8]. Defective DNA mismatch repair (dMMR) was assessed by testing for loss of MLH1, MSH2, MSH6 and PMS2 expression. The following antibodies were used: anti-MLH1 (M1), anti-MSH6 (44) (Ventana/Roche), anti-MSH2 (G2191129), and anti-PMS2 (EPR3947) (Cell Marque) and the immunohistochemical reactions were performed in a Benchmark XT (Ventana) automated immunohistochemical stainer according to the manufacturer’s instructions. Loss of MMR protein expression was defined as complete absence of nuclear staining in the presence of positive staining of stromal cells.

### Scoring

Immunohistochemical staining for each tumor core was independently assessed by two observers (PD and WD) who were blinded to the clinical and pathological data. In cases of disagreement, the result was reached by consensus. All tumor cells in the core of the tissue microarray were counted, and the percentage of tumor cell nuclei with unequivocal staining was recorded for each core. p21^WAF1^ expression was classified as negative (<1 % positive tumor nuclei, p21-) or positive (≥1 % tumor cells showing nuclear immunoreactivity, p21+). For assessement of p53 and TS expression, a histoscore system was used. In this system, which was proved useful and reproducible in assessing immunohistochemical staining, both average intensity and pattern scores were assessed [9, 10]. The proportion of positive cells was estimated and given a score on a scale from 1 to 6 (1 = 1 % to 4 %, 2 = 5 % to 19 %, 3 = 20 % to 39 %, 4 = 40 % to 59 %, 5 = 60 % to 79 %, and 6 = 80 % to 100 %). The average intensity of the positively stained tumor cells was given a score ranging from 0 to 3 (0 = no staining, 1 = weak staining, 2 = intermediate staining, and 3 = strong staining). A final score was then calculated by multiplying the percentage score by the intensity score, to yield a minimum value of zero and a maximum value of 18. A histoscore value of 7 was adopted as a cut-off for stratification of p53 expression into low (≤ 7, p53-) and high (>7, p53+), because the histogram of p53 values showed a local minimum at this point that clearly divided the study population into two subgroups. Similarly, stratification of TS expression into low (< 2, TS-) and high (≥ 2, TS+) was based on a local minimum in the histoscore histogram.

### Statistics

Associations between the tumor immunophenotype and other categorical variables were analyzed with Fisher’s exact test, whereas the Mann–Whitney test was used for associations with age. The Kaplan-Meier method was used for the univariate survival analysis, and the differences between compared groups were assessed by log-rank test. Cox’s proportional hazards model was used for univariate and multivariate analyses of factors associated with OS and DFS. The independent variables included in the model were age, gender, tumor site, Astler-Coller stage, histological grade, and the presence of expression of the tumor markers studied. A value of *P* < 0.05 was considered statistically significant. STATISTICA version 10 (StatSoft Inc., Tulsa, OK., USA) was used for the statistical analyses. This study has been carried out according to REMARK guidelines [11].

## Results

### TS, p53 and p21^WAF1^ expression, and correlation with clinico-pathologic parameters

**Fig. 1:**
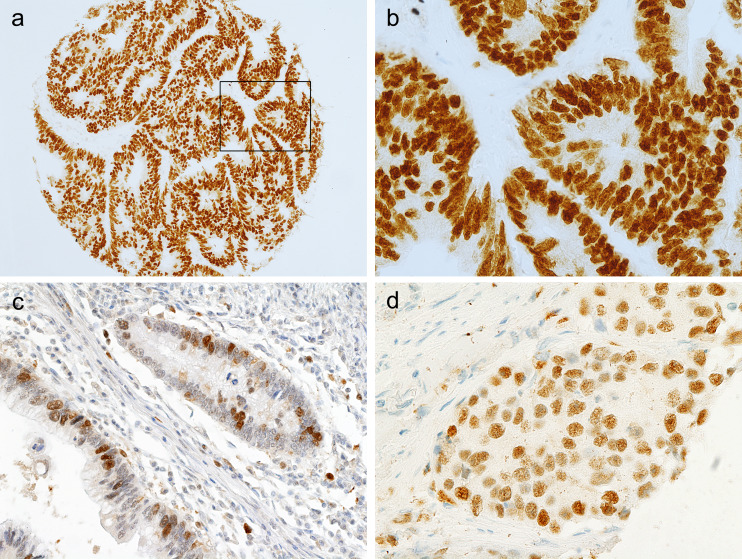
p53, TS and p21 immunostaining of representative colorectal cancer cores. **a** High expression of p53 in nuclei of tumor cells. **b** Marked fragment of the core from Fig. 1a at high magnification showing p53-positive (*brown*) nuclei in almost all cancer cells. **c** Low TS expression (*brown*) in nuclei of tumor cells. **d** p21 nuclear expression in tumor cells

**Fig. 2:**
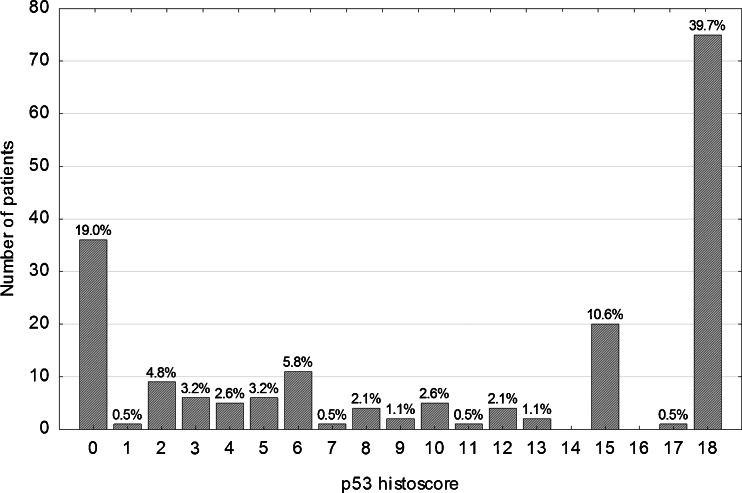
Distribution of p53 histoscores in the study group (*n* = 189)

**Fig. 3:**
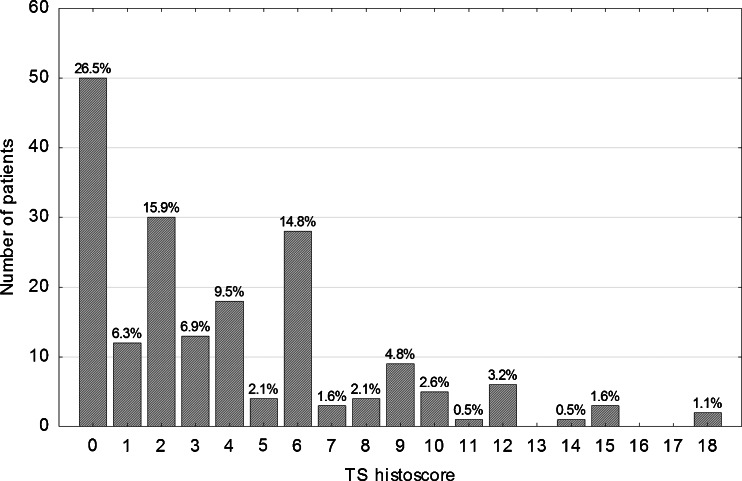
Distribution of TS histoscores in the study group (*n* = 189)

Immunohistochemical nuclear stainings were assessed using the anti-p53 (Fig. 1a and b), anti-TS (Fig. 1c) and anti-p21 (Fig. 1d) antibodies. High p53 (p53+) nuclear expression (histoscore p53 >7) was seen in 114 out of 189 samples (60.3 %). The distribution of the p53 histoscores is shown in Fig. 2. High TS (TS+) expression (histoscore TS ≥2) in tumor cell nuclei was noted in 127 out of 189 samples (67.2 %). The distribution of the TS histoscores is shown in Fig. 3. Nuclear expression of p21^WAF1^ (p21+) was noted in 126 out of 189 samples (66.7 %). The p21^WAF1^+/p53- immunophenotype was found in 50 out of 185 samples (26.5 %). Loss of MMR proteins was found in 31 out of 185 samples (16.8 %).

Next, the TS, p53, and p21^WAF1^ expression levels were correlated with the following parameters: age and gender of patients, tumor grade, stage and tumor site, and preoperative radiotherapy. High TS expression in tumor cell nuclei was seen more frequently in tumors located in the colon compared to those in the rectum (75.0 % versus 59.1 %, *P* = 0.03). High p53 expression was seen more frequently in males than in females (68.6 % versus 50.6 %, *P* = 0.02). Expression of p21^WAF1^ was seen more frequently in stage C than in stage B2 CRCs (72.6 % versus 57.9 %, *P* = 0.04). The p21^WAF1^+/p53- immunophenotype was seen more frequently in females than in males (34.5 % versus 19.6 %, *P* = 0.03). No other statistically significant associations were found.

### Survival of patients with CRC defined by p21^WAF1^/p53 immunophenotype and TS expression

Univariate analysis in all patients (stage B2 + C) revealed that high TS expression was associated with a significantly worse OS (*P* = 0.038, HR = 2.26, 95 % CI 1.05–4.87). The association with DFS did not reach significance (*P* = 0.082, HR = 1.59, 95 % CI 0.94–2.68). p21^WAF1^ expression was associated with a significantly better OS (*P* = 0.0076, HR = 0.44, 95 % CI 0.24–0.81) and a borderline better DFS (*P* = 0.076, HR = 0.66, 95 % CI 0.41–1.05), while high p53 expression was associated with a significantly worse DFS (*P* = 0.029, HR = 1.74, 95 % CI 1.06–2.87). The association with OS did not reach significance (*P* = 0.099, HR = 1.73, 95 % CI 0.90–3.32). The p21^WAF1^+/p53- (i.e., p21^WAF1^ expression and low p53) immunophenotype was found to be associated with both a significantly better OS (*P* = 0.0057, HR = 0.23, 95 % CI 0.08–0.65) and a significantly better DFS (*P* = 0.0023, HR = 0.36, 95 % CI 0.18–0.69). For combined phenotypes other than p21^WAF1^+/p53- (i.e., p21^WAF1^+/p53+, p21^WAF1^-/p53+, and p21^WAF1^-/p53-, collectively defined as P&P, *n* = 139), univariate analysis revealed a significant association of high TS expression with a worse DFS and OS (*P* = 0.02, HR = 1.90, 95 % CI 1.09–3.32 and *P* = 0.009, HR = 2.99, 95 % CI 1.32–6.80, respectively).

Loss of MMR protein expression was found to be associated with a better DFS (*P* = 0.01, HR = 0.32, 95 % CI 0.13–0.79), but not a better OS (*P* = 0.13). Multivariate analysis involving sex, age, Astler-Coller stage, tumor grade, tumor site, TS expression, the p21^WAF1^/p53 immunophenotype and loss of MMR protein expression identified Astler-Coller stage, high TS expression and the p21^WAF1^+/p53- immunophenotype as independent factors associated with DFS and OS (Table 2). In this analysis, the association of loss of MMR protein expression with DFS did no longer reach statistical significance.

**Table 2:** Multivariate analysis of disease-free (DFS) and overall (OS) survival of stage B2 and C colorectal cancer patients (*n* = 185) treated with 5FU-based adjuvant chemotherapy

Parameters	DFS		OS	
	Hazard ratio (95 % CI)	*P*	Hazard ratio (95 % CI)	*P*
Male Sex	1.27 (0.77–2.09)	0.36	1.39 (0.73–2.68)	0.32
Age	1.01 (0.93–1.03)	0.60	1.00 (0.97–1.03)	0.98
Astler-CollerC	2.46 (1.43–4.25)	**0.001**	2.69 (1.28–5.61)	**0.009**
Grade 3	1.51 (0.93–2.46)	0.09	1.94 (1.01–3.70)	**0,046**
Site Rectum	0.97 (0.61–1.64)	0.99	0.85 (0.45–1.61)	0,61
High TS	1.73 (1.01–2.95)	**0.045**	2.39 (1.09–5.23)	**0.03**
p21+p53-*	0.36 (0.18–0.72)	**0.004**	0.19 (0.07–0.57)	**0.003**
MMRp loss**	0.41 (0.16–1.06)	0.07	0.71 (0.24–2.11)	0.54

* p21 + p53- = expression of p21^WAF1^ and low expression of p53. ^**^MMRp = mismatch repair protein

**Fig. 4:**
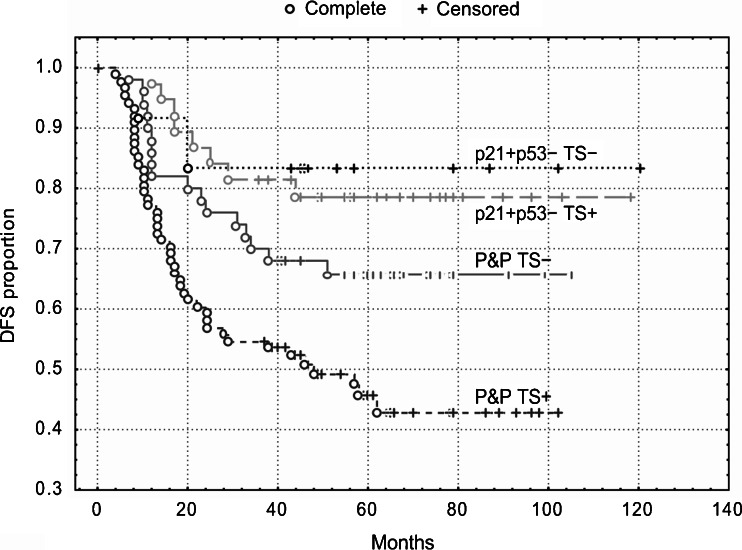
DFS of patients with stage B2 + C colorectal carcinomas (*n* = 189) categorized according to TS expression. TS stratifies patients with P&P immunophenotype (i.e., p21+/p53+, p21-/p53-and p21−/p53+): P&P TS + versus P&P TS-curves, *P* = 0.02. The P&P TS + curve differs also from other curves, i.e., from the p21+/p53−TS + curve (*P* = 0.0004) and from the p21+/p53−TS− curve (*P* = 0.02). TS does not significantly stratifiy patients with a p21+/p53− immunophenotype (*P* = 0.80). p21 = p21^WAF1^

**Fig. 5:**
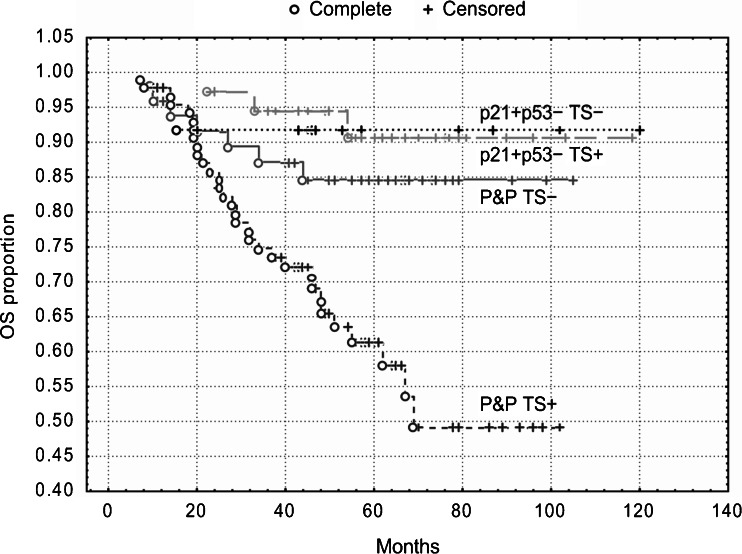
OS of patients with stage B2 + C colorectal carcinomas (*n* = 189) categorized according to TS expression. TS stratifies patients with a P&P immunophenotype (i.e., p21+/p53+, p21−/p53− and p21−/p53+): P&P TS + versus P&P TS− curves, *P* = 0.005. The P&P TS + curve differs also from other curves, i.e., from the p21+/p53-TS + curve (*P* = 0.0003) and from the p21+/p53−TS− curve (*P* = 0.05). TS does not significantly stratifiy patients with a p21+/p53− immunophenotype (*P* = 0.85). p21 = p21^WAF1^

Kaplan-Meier survival curves showed that patients with the p21^WAF1^+/p53- immunophenotype exhibited the best DFS and OS (Figs. 4 and 5), but TS expression did not stratify these subgroups (*P* = 0.80 and *P* = 0.85, respectively). However, TS expression did stratify patients with the P&P phenotype into two subgroups, which differed with respect to DFS and OS (Figs. 4 and 5). The P&P TS + subgroup was associated with a worse DFS (*P* = 0.02) and OS (*P* = 0.005) compared to the P&P TS- subgroup. The P&P TS + curve also differed significantly also from other curves, i.e., from the p21^WAF1^+/p53-TS + curve and from the p21^WAF1^+/p53- TS- curve (Figs. 4 and 5), with respect to DFS (*P* = 0.0004 and *P* = 0.02, respectively) and OS (*P* = 0.0003 and *P* = 0.05, respectively). These findings were confirmed in a multivariate analysis, which revealed an association of the P&P TS + immunophenotype with a worse DFS (*P* = 0.0009, HR = 2.27, 95 % CI 1.39–3.68) and OS (*P* = 0.0002, HR = 3.82, 95 % CI: 1.89–7.73) compared to all other immunophenotype combinations.

There are differences in therapeutic approaches between cancers of the colon and the rectum. In order to assess whether our results might depend on the site of the tumor, patients with tumors localized in the colon and the rectum were analyzed separately.

#### Colon

**Fig. 6:**
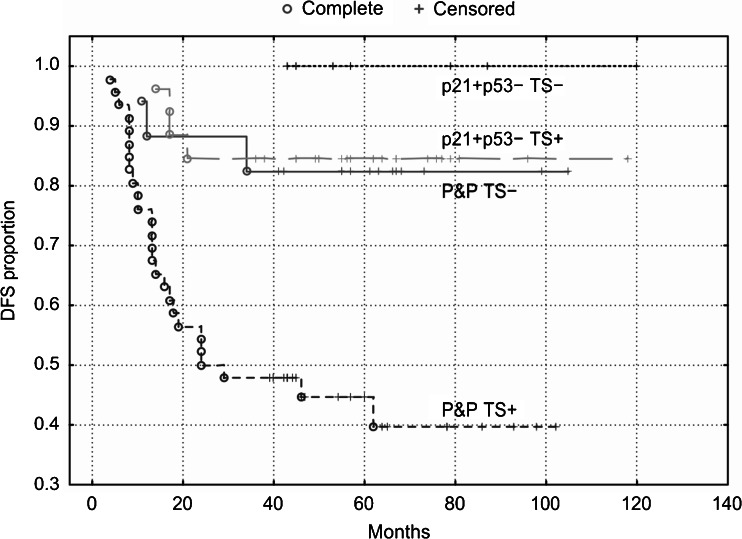
DFS of patients with stage B2 + C colon carcinoma (n = 96) categorized according to TS expression. TS stratifies patients with a P&P immunophenotype (i.e., p21+/p53+, p21−/p53− and p21−/p53+): P&P TS + versus P&P TS− curves, *P* = 0.006. The P&P TS + curve differs also from other curves, i.e., from the p21+/p53-TS + curve (*P* = 0.0005) and from the p21+/p53−TS− curve (*P* = 0.007). TS does not significantly stratifiy patients with a p21+/p53− immunophenotype. p21 = p21^WAF1^

**Fig. 7:**
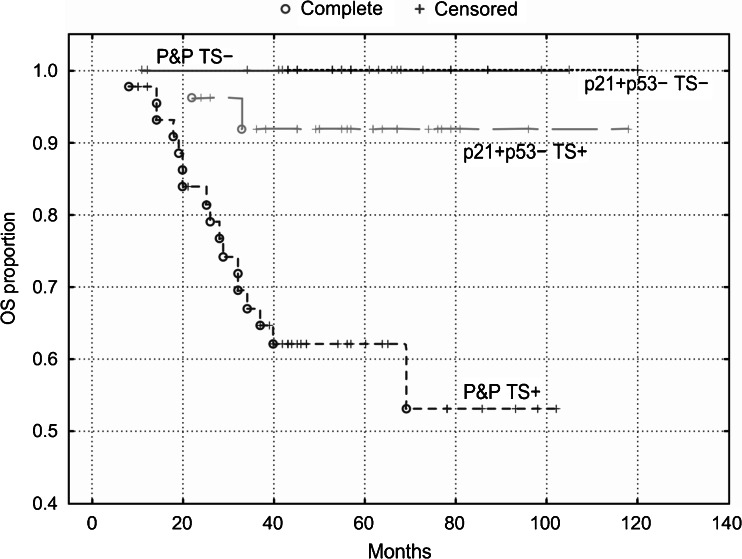
OS of patients with stage B2 + C colon carcinoma (*n* = 96) categorized according to TS expression. TS stratifies patients with a P&P immunophenotype (i.e., p21+/p53+, p21−/p53− and p21−/p53+): P&P TS + versus P&P TS− curves, *P* = 0.005. The P&P TS + curve differs also from other curves, i.e., from the p21+/p53-TS + curve (*P* = 0.005) and from the p21+/p53− TS− curve (*P* = 0.003). TS does not significantly stratifiy patients with a p21+/p53-immunophenotype. p21 = p21^WAF1^

Similar to the overall group of patients, we found that TS expression did not significantly stratify patients with a p21^WAF1^+/p53- immunophenotype, which was associated with the best DFS and OS. However, TS expression did stratify patients with tumors exhibiting the P&P phenotype localized in the colon into two subgroups, which differed with respect to DFS and OS (Figs. 6 and 7). The P&P TS +  subgroup was associated with a worse DFS (*P* = 0.006) and OS (*P* = 0.005) compared to the P&P TS- subgroup. The P&P TS +  curve differed significantly also from other curves, i.e., from the p21^WAF1^+/p53- TS +  curve and from the p21^WAF1^+/p53- TS- curve (Figs. 6 and 7), with respect to DFS (*P* = 0.0005 and *P* = 0.007, respectively) and OS (*P* = 0.005 and *P* = 0.003, respectively). These findings were confirmed in multivariate analyses, which showed an association of the P&P TS + immunophenotype with a worse DFS (*P* = 0.0004, HR = 4.93) and OS (*P* = 0.002, HR = 10.55) (Table 3) compared to all other immunophenotype combinations. This association remained significant (DFS: *P* = 0.0008, HR = 4.80, 95 % CI 1.92–12.03; OS: *P* = 0.004, HR = 8.82, 95 % CI 1.99–39.03) when cases with loss of MMR protein expression were excluded from the analysis.

**Table 3:** Multivariate analysis of disease-free (DFS) and overall (OS) survival of stage B2 and C colon cancer patients (*n* = 94) treated with 5FU-based adjuvant chemotherapy

Parameters	DFS		OS	
	Hazard ratio (95 % CI)	*P*	Hazard ratio (95 % CI)	*P*
Male Sex	1.24 (0.58–2.64)	0.58	1.68 (0.61–4.58)	0.32
Age	1.01 (0.98–1.05)	0.53	1.01 (0.96–1.06)	0.74
Astler-Coller C	2.28 (1.03–5.07)	**0.04**	1.76 (0.67–4.68)	0.25
Grade 3	1.63 (0.79–3.36)	0.19	1.08 (0.42–2.79)	0.88
P&P/TS+	**4.93** (2.03–11.93)	**0.0004**	**10.55** (2.36–47.30)	**0.002**
MMRp loss*	0.37 (0.08–1.65)	0.19	0.33 (0.04–2.63)	0.88

*MMRp = mismatch repair proteins

#### Rectum

In patients with rectal cancer, (*n* = 93) no significant association was found between the P&P/TS+ immunophenotype and DFS (*P* = 0.29 for univariate and *P* = 0.41 for multivariate analysis, respectively) or OS (*P* = 0.07 for univariate and *P* = 0.23 for multivariate analysis, respectively). The percentage of tumors exhibiting the P&P/TS+ immunophenotype did not differ significantly between patients who did or did not undergo preoperative radiotherapy (56.3 % versus 52.5 % respectively, *P* = 0.82). There was significant interaction between the site of the tumor (colon versus rectum) and the P&P/TS+ immunophenotype with respect to an association with DFS (*P* = 0.01) and OS (*P* = 0.03) in a multivariate analysis. These findings indicate that only patients with colon cancer (but not those with rectal cancer) treated with adjuvant 5FU could be stratified into better or worse prognostic subgroups by TS expression when the p21^WAF1^/p53 immunophenotype was considered.

## Discussion

5FU-based adjuvant chemotherapy reduces the risk of recurrence by 41 % and the overal death rate by 33 % of stage C CRC patients [12]. In stage II CRC, this type of chemotherapy improves the 5-year survival rate by a mere 3–6 % [13]. In advanced CRC improvements in survival obtained with various treatments (e.g. 5FU plus leucovorin, 5FU plus methotrexate) have remained limited, even in cases where initial response rates were substantially improved [14, 15]. Hence, it appears that a sizable percentage of patients will not benefit from 5FU-based adjuvant chemotherapy, but neverthesless will be exposed to the toxic effects of this treatment [16]. Therefore, it is important to better define subgroups of CRC patients who will benefit from 5FU-based treatment and those who will not. Previously, we have shown that expression of p21^WAF1^ in colorectal tumor cells identifies a subgroup of Astler-Coller stage B2 patients who may significantly benefit from 5FU-based chemotherapy. This, thus, allows a better selection of patients for adjuvant chemotherapy [17]. In this report, we show that TS expression analysis, in conjunction with p21^WAF1^/p53 immunophenotyping, allows the identification of prognostic subgroups of CRC patients subjected to 5FU-based adjuvant chemotherapy.

We found a high nuclear TS expression in 67.2 % of the CRC samples, which is in agreement with the findings of Wong et al. (69 %) [18]. TS is one of the principle enzymes involved in DNA synthesis, and serves as a molecular target of 5FU [19]. TS also exhibits properties of an oncogene [20]. The multidirectional mechanism of action of 5FU includes an inhibition of TS and a direct interference with DNA and RNA synthesis through incorporation of 5-fluoronucleotides [2]. In vitro studies using CRC cell lines have suggested a predictive importance of TS expression with respect to the efficacy of 5FU-based chemotherapy [21]. However, clinical trials assessing the predictive/prognostic significance of TS expression in CRC patients have reported discrepant findings. Meta-analyses revealed a moderately negative impact of high TS expression on the survival of patients with CRC [22], and a slightly negative correlation of TS expression with a response to 5FU [23]. It was concluded that, in clinical practice, TS expression alone could not be used as a predictive marker [23]. Hence, despite the well-documented role of TS as a target for 5FU-based therapy, it failed to enter the clinic as a useful predictive marker for the response to 5FU (for literature review see [24–26]). Using CRC cell lines, it has been suggested that resistance to 5FU chemotherapy cannot be assigned solely to TS or p53 expression [27]. Recent data indicated a need for including cell cycle-associated parameters in the search for predictive markers beneficial for 5FU-based chemotherapy in CRC [6, 28]. It seems, therefore, reasonable to assume that several factors, rather than one (i.e., TS), should be considered in order to identify CRC subgroups sensitive to 5FU treatment.

Through multivariate analysis we observed no significant association between TS expression alone and DFS or OS. However, when the p21^WAF1^/p53 phenotype was taken into account, it was only in the subgroup of patients with colon cancer and an immunophenotype other than p21^WAF1^+/p53- that TS expression emerged as an important prognostic/predictive marker. High TS expression in a subgroup of patients with colon cancer characterized by phenotypes other than p21^WAF1^+/p53- was associated with a poor DFS and OS. Thus, the expression of these markers may provide prognostic/predictive information on the outcome of 5FU-based chemotherapy. Considering the subgroup of patients with colon cancer exhibiting phenotypes other than p21^WAF1^+/p53-, it seems that those with a low TS expression may more likely benefit from 5FU-based adjuvant therapy, whereas those with a high TS expression may not benefit because they already exhibit poor DFS and OS rates, despite having received 5FU-based therapy. On the other hand, TS expression did not stratify patients with colon cancer characterized by the best prognostic immunophenotype, i.e., p21^WAF1^+/p53-, with respect to either DFS or OS. Hence, for this subgroup of patients TS expression should not be expected to act as a prognostic/predictive marker for 5FU-based adjuvant therapy.

Patients with rectal cancer treated with adjuvant 5FU could not be stratified into better or worse prognostic subgroups by TS expression when the p21^WAF1^/p53 immunophenotype was considered. One cannot exclude the possibility that pre- and postoperative radiotherapy may affect the results beyond p21^WAF1^/p53 and TS expression. It could also downstage rectal cancer and/or the result may be site-specific. Currently, this problem remains unresolved and requires further study on larger groups of patients.

Several clinical studies have focused on associations of the p21^WAF1^/p53 immunophenotype alone with survival, with discrepant results [29–33]. In agreement with our study, several investigators have reported the shortest survival for patients with p21^WAF1^-/p53+ tumors and the longest for patients with p21^WAF1^+/p53- tumors [31, 32]. However, others reported the best prognosis for low p21^WAF1^ expression combined with high p53 expression [29, 33]. Ropponen et al. [30] failed to show a correlation between the p21^WAF1^/p53 immunophenotype and survival. These discrepancies may, at least partially, be attributed to the heterogenous groups of patients studied, i.e., the stage and type of adjuvant therapy, as well as the small sizes of the subgroups in the studies. However, they may also reflect the multifunctional activity of the p53-p21^WAF1^ cell cycle checkpoint pathway [34]. It is well known that p53 and its downstream target p21^WAF1^ are essential for maintaining genomic stability. Our results suggest that the effect of 5FU-based treatment of patients with CRC may depend on the status of the p53-p21^WAF1^ pathway. Patients with tumors with a functional p53-p21^WAF1^ pathway (p21^WAF1^+/p53-) exhibited the best survival, irrespective of TS expression (i.e., presumably irrespective of 5FU-based adjuvant therapy–which is directed against TS–since the therapy could not improve the prognosis of this a priori best prognostic subgroup). In other words, patients in this subgroup could not benefit from 5FU-based adjuvant therapy because they have already had the best prognosis. On the other hand, if the p53-p21^WAF1^ pathway was impaired (presumably in the P&P subgroup comprising p21^WAF1^+/p53+, p21^WAF1^-/p53+ or p21^WAF1^-/p53- tumors) then only survival of patients with tumors exhibiting low TS expression could be improved by this type of adjuvant treatment.

In our study, both the p21^WAF1^/p53 immunophenotype and loss of MMR protein expression were found to act as prognostic indicators for DFS in univariate analyses. However, only the former retained statistical significance in a multivariate analysis. Therefore, the p21^WAF1^/p53 immunophenotype seems to be a better predictor of DFS than loss of MMR protein expression in patients with stage B and C Astler-Coller CRC subjected to adjuvant 5FU-based therapy. Similarly, although both mutant *TP53* and MSI-H were found to be prognostic indicators for DFS in univariate analyses, only *TP53* retained statistical significance in multivariate analyses [35]. These results further underscore the importance of the p53-p21^WAF1^ cell cycle checkpoint pathway for the prognosis of patients with CRC treated with 5FU-based therapy.

We would also like to mention some limitations of this study. First, this is a retrospective study and the series of patients is rather small. Therefore, our results should be confirmed by further prospective randomized trials. Second, the choice of cut-off levels requires a short comment. For p21^WAF1^ we used a cut-off level of ≥1 %. In line with our previous report [17], such a cut-off for a positive p21^WAF1^ expression was associated with a better DFS and OS of patients with CRC. Since there are no generally accepted cut-off levels for p53 or TS protein nuclear expression, we tested several local minima (present on histograms) and we have chosen those which gave the strongest associations with survival. Various threshold levels have been used in the assessment of immunohistochemical p53 expression in CRC. In 14 reports on the prognostic significance of immunohistochemical assessment of p53 accumulation in CRC, cut-off levels ranging from >0 % to >20 % have been applied and, consequently, the percentage of p53 positive tumors ranged from 30 % to 63 % [36]. In other reports cut-off levels of 25 % [37], >50 % [38] or ≥50 % [39] have been used, yielding 63 %, 48.5 % and 43 % of p53 positive tumor cells, respectively. With our cut-off level (histoscore p53 > 7), high p53 (p53+) nuclear expression was seen in 60.3 % of the samples, which is within the range found in the literature. A number of different scoring methods and threshold levels have been reported for the assessment of immunohistochemical TS expression in colorectal cancer: (1) The intensity of TS staining of tumors was arbitrarily graded from 0 to 3 (or 0 to 4) and dichotomized (grades 0 to 1 defined as low level, and 3 to 4 as high level of TS expression) – a review of 13 studies [22] and [24, 26, 40, 41], (2) TS expression was dichotomized using arbitrary thresholds of 10 %, 15 %, 20 % [22] or ≥30 % [42] of stained cells per field, (3) Histoscores were calculated [18, 43]. In these reports the proportion of cases expressing high levels of TS ranged from 14 % to 80 %. We found 67.2 % of CRCs exhibiting high TS expression, which is again within the range found in the literature.

Currently, it is not known what percentage of tumor cells has to be positive for those markers to affect the biology of a tumor in such a way that it can influence the survival of patients subjected to adjuvant 5FU-based therapy. Therefore, cut-off levels are usually chosen empirically (e.g. 5 %, 10 %, 20 %, 50 %). However, it seems that a histoscore (which we applied in this study) better reflects biologic associations than a mere percentage count. Wong et al. [18] showed that assessment of TS expression with the use of a histoscore showed statistical differences between patients with CRC responding and non-responding to 5FU-based therapy, whereas when only the percentage of positively stained nuclei was assessed no differences were found. In that report a median histoscore was used as a threshold level. In our study we used a cut-off close to median. Interestingly, Munro et al. [44] in a review based on 168 reports, including survival data of 18.766 patients, assessing p53 abnormalities and clinical outcomes in colorectal cancer found “no evidence for any relationship between the criterion used to define ‘positive’ by immunohistochemistry and outcome”. They noted, for example, that the absolute survival rate difference was 13.4 % when the criterion was set at >1 % cells positive, and 13.5 % when a cut-off value of >10 % positivity was used.

The major advantage of immunohistochemistry on tissue microarrays is that a large number of tumors can be assessed simultaneously under identical laboratory conditions, which improves the reproducibility of the results. A limitation of this technology, however, is that small cores transferred from donor to recipient paraffin blocks may not be representative of the entire tumor. Therefore, sampling a typical and representative region of a tumor is the most important step in tissue microarray construction. Here, we employed one core from a carefully identified, histologically relatively homogenous, representative area exhibiting the highest mitotic activity at the outer invasive front of each CRC. With this approach, we found high expression of p53 in 60.3 %, high TS expression in 67.2 %, and p21^WAF1^ expression in 66.7 % of CRCs, which is within the range reported for these proteins in the literature (30 %–63 % for p53, 15–80 % for TS and 16 %–87 % for p21^WAF1^).

There are divergent opinions on the number of cores required to establish associations between biomarkers and clinicopathological parameters. Hoos et al. [45] reported that correlations between phenotypes and clinical outcome did not significantly differ between full sections and triplicate 0.6–mm core tissue microarrays. These correlations were, however, not significantly different when only one 0.6-mm core tissue microarray was used [46, 47]. Zhang et al. [46] reached >97 % concordance rates between tissue microarrays and full sections for several markers, and they concluded that “a tissue microarray with a single core per specimen ensures full biological representativeness to identify associations between biomarkers and clinicopathological parameters, with no significantly associated sampling bias”. Clearly the lower the heterogeneity of the tumor cells with respect to the proteins under study, the better the concordance of tissue microarrays with the whole sections. Wong et al. [18] tested intratumoral variation of TS nuclear expression in CRCs and found it to be rather small (maximum difference in TS histoscore of 15 when a range from 0 to 300 was applied). Barely noticeable intratumor heterogeneity of TS expression was noted in another report [42]. However, the key step in the construction of tissue microarrays remains careful sampling of representative tumor regions. According to Zhang et al. [46] “The biopsy of 0.6 mm in diameter taken from a typical and representative region of the tumor provides a reliable and efficient system for large-scale analysis of cancer tissues on tissue microarray platforms and is useful for large-scale clinicopathological studies, conserving the molecular profile of the molecular markers that are clinically relevant”.

**Fig. 8:**
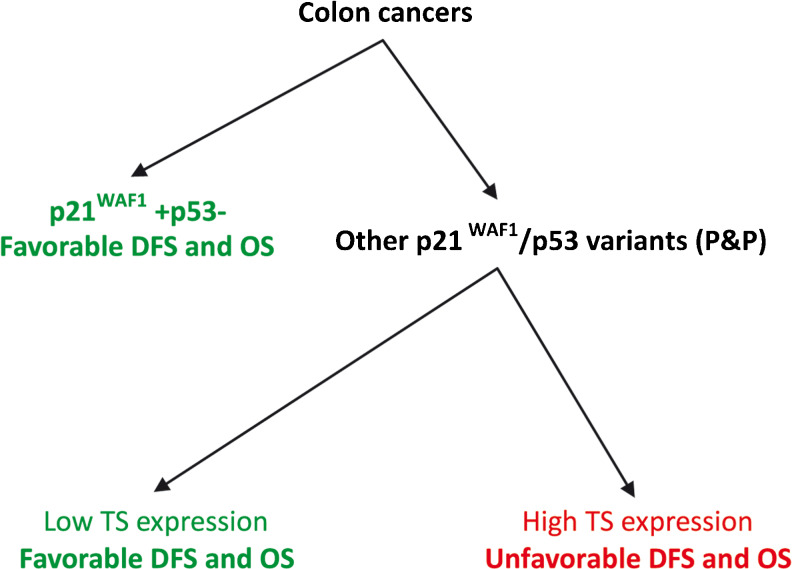
Schematic algorithm for determining the prognostic significance of nuclear TS expression in patients with stage B2 + C colon cancer subjected to 5FU-based adjuvant chemotherapy

We conclude that (1) the p21^WAF1^+/p53- immunophenotype is associated with a favorable prognosis in patients with stage B2 + C colon cancer treated with 5FU-based chemotherapy, regardless of the level of TS expression, (2) TS expression stratifies patients with colon cancer exhibiting immunophenotypes other than p21^WAF1^+/p53- and treated with 5FU in two subgroups characterized by a worse (high TS expression) and a better (low TS expression) prognosis. The observed strong association of the P&P TS+ immunophenotype with a worse DFS in both univariate and multivariate analyses suggests the predictive significance of TS expression for 5FU-based adjuvant therapy in patients with colon cancer exhibiting the P&P immunophenotype. Finally, (3) the results of this study suggest that future clinical trials evaluating the predictive significance of TS expression in colon cancer for 5FU-based adjuvant chemotherapy should take the p21^WAF1^/p53 immunophenotype of the tumor cells into account. Therefore, we propose a summarizing algorithm (depicted in Fig. 8) as a starting point for clinical trials assessing the predictive significance of TS expression in patients with stage B2 + C colon cancer subjected to 5FU-based adjuvant chemotherapy. Since this is a retrospective study, our results should be confirmed in additional prospective randomized studies.
